# Reduction mammoplasty

**Published:** 2008-10

**Authors:** Shrirang Purohit

**Affiliations:** Lilavati Hospital, Mumbai - 400 050, India

Reduction Mammoplasty is a procedure in which a volumetric reduction of the breast is done. In the process it also improves the shape of the breast and repositions the nipple areola complex.

## HISTORICAL PERSPECTIVES

Concern with excessively large breasts in males is documented as early as the time of Paulus of Aegina (635-90).[1]

The earliest recorded procedures to reduce overly large breasts in females were developed by reconstructive and aesthetic surgeons in the last two decades of the 19^th^ century. These developments paralleled similar developments in aesthetic nasal surgery.

As with the nose, breast reduction surgery had its origin in an effort to change the external features to make a person more racially acceptable.

Breast size was also thought to be an important determinant of the social class to which a person belonged and efforts to change or reduce the breast size were to help assimilation in a particular social class, especially in countries like Brazil, which have a cultural and racial mix in society.[2]

The first 19^th^ century approaches suggested by Theodor Billroth[3] and Alfred Pousson[4] did not concern themselves with the aesthetics at all; removal rather than reduction was the procedure of choice.

It was only in the late nineteenth century that the concept of a “natural breast” gradually evolved.

Theodore Galliard –Thomas suggested a sub-mammary incision to rescue at least part of the glandular disc.[2]

Vincenz Czerny transplanted the nipple following a simple mastectomy to preserve the natural breast.[2]

It was only in the first decade of the twentieth century that Hippolyte Morestin[5] and Eugene Hollander[6] undertook aesthetic breast reduction and described a lateral glandular resection technique with a L shaped oblique closure.

By the1930s, however, breast reduction entirely crossed out of the realm of reconstructive surgery into that of aesthetic surgery.

Lexer described his procedure of inferior glandular resection with subcutaneous undermining and nipple transposition in 1921[7] and the same was later described by Kraske in 1923[8] and the procedure came to be known as the Lexer-Kraske operation.

Axhausen[9] pioneered his three step technique:

1. Extensive subcutaneous undermining of the breast to reduce the glandular portion of the breast.
2. Nipple transposition
3. Fashioning of a skin brassiere

Thorek[10] was the first to advocate free nipple grafting in cases of pronounced macromastia.

Biesenberger[11] combined three elements:

- Separation of skin from gland.
- Resection of lateral half of the gland.
- Transposition of nipple on the retained gland.

Schwarzmann[12] had described a procedure where the nipple and areola was transposed, based on a medial pedicle. This was a major advance where the blood supply of the nipple areola was based on a dermal pedicle

Gillies and McIndoe[13] removed breast tissue above the areola and approximated the edges.

Aufricht[14] removed a wedge from the upper quadrant, the upper and lateral quadrants or the entire upper half.

Wise[15,16] used a modified Biesenberger operation but his contribution was more in the form of excision patterns and mechanical aids to produce a safer reduction.

Strombeck in 1960[17] published his article describing a pattern and reduction technique with transposition of nipple and areola on a transverse bipedicled flap of glandular tissue. 10 years later, Strombeck showed that the same flap of glandular tissue could be safely unipedicled as well.

Pitanguy[18] described a horizontal dermal pedicle with a keel shaped resection of the central and inferior glandular portion. In 1967, he described the superior dermal pedicle.[19]

Dufourmental and Mouly[20] described the lateral wedge mammoplasty to reduce medial scarring.

Skoog[21] described his lateral dermal pedicle, his major contribution being the suggestion of transposing the nipple in the breast meridian.

McKissock[22] described the popular vertical bipedicle dermal pedicle technique where the vascularity of the nipple areola depended on the intact dermal parenchymal pedicle.

The B technique of Regnault also attempts to reduce medial scarring.[22]

Robbins,[23] Courtiss and Goldwyn,[24] all contributed to the development of the inferior pedicle technique.

Central breast pedicle with circumferential resection around it was described by Hester.[25]

Courtiss[26,27] in a series of articles suggested liposuction alone as a means of reducing breast volume.

Breast reduction with vertical short scars only has been a much vaunted goal in breast reduction surgery and Arie,[28] Lassus,[29–31] Madeleine Lejour[32] and Hall-Findlay[33,34] have all made significant contributions to the effort.

### Requirements of an ideal breast reduction

These have been put forth by Biesenberger[11] and have stood the test of time,

1. The breast should be lifted to a youthful and natural form in proportion to other parts of the body.
2. The two breasts should be symmetrical
3. The nipple and areola should be translocated to an appropriate location.
4. The blood supply to nipple and areola should not be jeopardized.
5. The function of the breast should be preserved.
6. The scars should not be visible through normal clothing or be above the areola.
7. The operation must be applicable to all forms of deformity.
8. The procedure should be a one stage operation.

## PATIENT PROFILES[35]

### Teenagers

Giant virginal hypertrophy is a condition in which young girls around puberty develop massive breasts which are out of proportion to the rest of their body. These patients suffer major psychological problems and are a focus of cruel jokes, do not fit into normal clothes and also find it difficult to take part in athletic activities. They also experience chronic shoulder, back and breast pain.

These patients are good candidates for reduction, however they must be cautioned that they may require repeat surgery if the breasts increase in size as they grow older.

Over reduction should be avoided. Attention must be paid to the possibility of retaining lactation function and nipple-areola sensation in designing the pedicle. When a free nipple graft is unavoidable, the patient and relatives must be counselled thoroughly in advance.

### Women after childbearing

These patients are usually interested more in correction of the post-lactation ptosis, also desire fullness in the empty upper breast pole and relief from symptoms due to heavy breasts.

Some of these can be treated by liposuction alone if the breasts are found to be predominantly fatty. Close attention must be paid to skin tone and elasticity before the option of liposuction alone is given to the patient.

### Women after menopause

These patients usually require breast reduction to relieve the symptoms related to large and heavy breasts. These usually request large reductions in cup size.

## SURGICALLY RELEVANT ANATOMY[36]

The most important aspect of the anatomy is the understanding of the blood supply and nerve supply to the nipple areola complex. The Internal Mammary artery, Intercostal arteries and the Lateral Thoracic artery supply the breast.

The internal mammary artery is responsible for about 60% of the blood supply to the breast, the medial or superomedial pedicle being based on the anterior perforating branches of this vessel especially the 2nd and 3^rd^, which anastomose with the branches of the Lateral Thoracic artery.

The lateral thoracic artery supplies about 30% of the blood supply to the breast, the branches course inferomedially and anastomose with the branches of the Internal Mammary and Intercostal arteries.

The Intercostal arteries, 3^rd^ 4^th^ and 5^th^ are the least important of the arteries supplying the breast. The 4^th^ and 5^th^ are responsible for the viability of the inferior pedicle.

Sensory nerve supply to the nipple areola complex comes from the 4^th^ lateral intercostals branch, which enters laterally through the 4^th^ interspace and runs medially under the deep fascia for a few cms. It then courses upwards through the breast tissue to supply the nipple-areola. Some sensation is also provided by the 3^rd^ and 5^th^ lateral intercostal branches in the lateral breast area and the 3^rd^ to 5^th^ anterior branches of the intercostal nerves.

### Procedures

While planning breast reduction, it is important to understand that the skin resection pattern and the placement of the pedicle are two entirely different things.

Most of the skin resection patterns can be combined with most pedicles.

However the inferior pedicle has been most associated with an Inverted T skin resection and a Superior Pedicle or superomedial pedicle with a vertical skin resection.

## PEDICLES

### Horizontal bipedicle

This was the Strombeck pattern with the blood supply coming from both sides, the dermal pedicle was sufficient to maintain nipple areola viability but caused nipple retraction and inclusion of glandular element in the pedicle made insetting difficult.[17]

### Vertical bipedicle

Mckissock's Vertical Bipedicle was very popular for a long time as it provided good blood supply and also was easy to inset.[22]

### Inferior pedicle

**Diagram 1:**
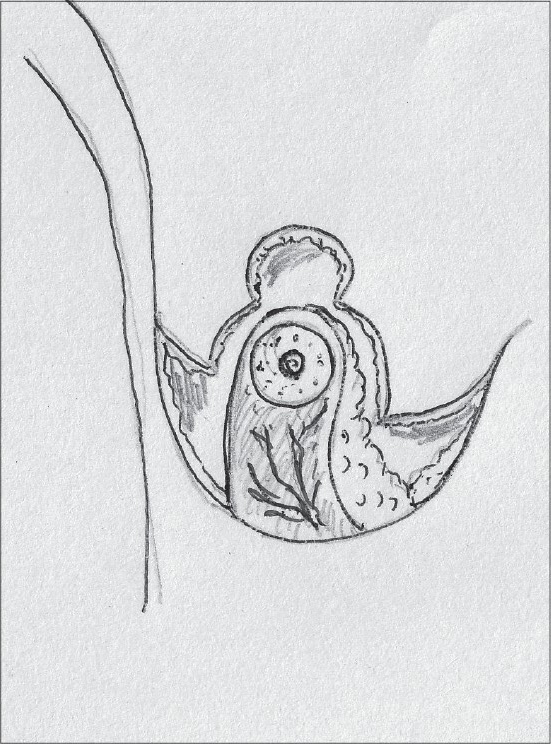
Blood supply of the inferior pedicle

Inferior pedicle proved to be sufficient to sustain the nipple areola complex and also had other advantages – good circulation, good sensation and possibility of breast-feeding. As a result, it replaced the vertical bipedicle [Diagram 1].[23,24]

### Superior pedicle

**Diagram 2:**
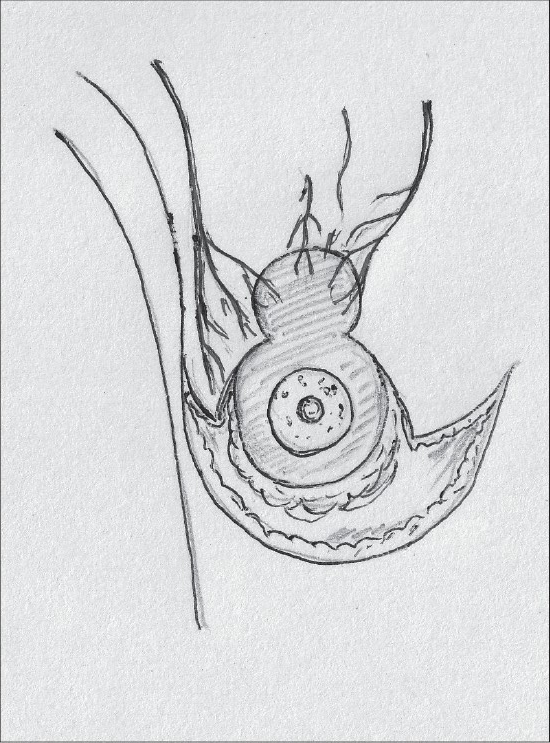
Blood supply of the superior pedicle

Superior pedicle has good circulation but is not very easy to inset and has to be thinned for better inset. Being a dermal pedicle breast feeding is no longer possible [Diagram 2].[36]

### Central pedicle

**Diagram 3:**
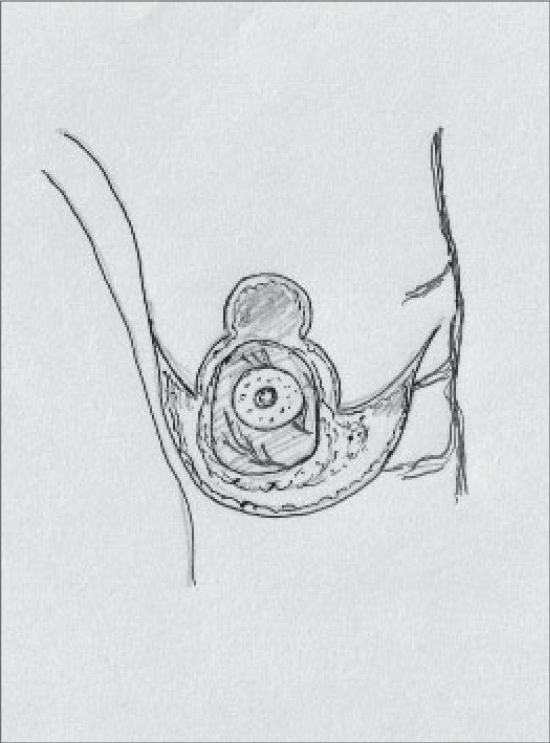
Blood supply of the central pedicle

The Central pedicle is a modification of the Inferior pedicle with the removal of the dermal bridge. The blood supply is the same - perforating branches of the intercostal arteries [Diagram 3].[25]

The venous drainage follows the artery so a dermal bridge is not required. However one must be very careful about shear injuries to the pedicle at its base on the pectoral muscles.

It preserves sensation and breast feeding potential.

### Lateral pedicle

**Diagram 4:**
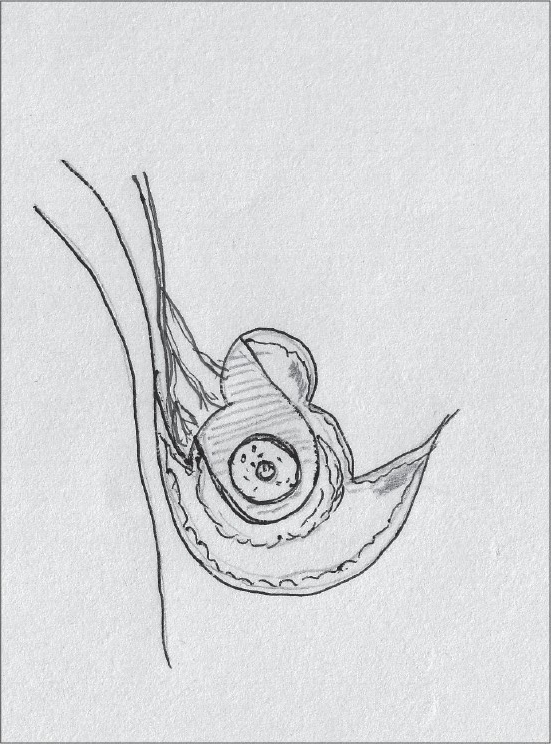
Blood supply of the lateral pedicle

This pedicle is half the pedicle of Strombeck's method and it is easier to inset, also has good viability and is based on the lateral thoracic artery perforators; this pedicle is not as commonly used as the rest [Diagram 4].[21]

### Medial pedicle

**Diagram 5:**
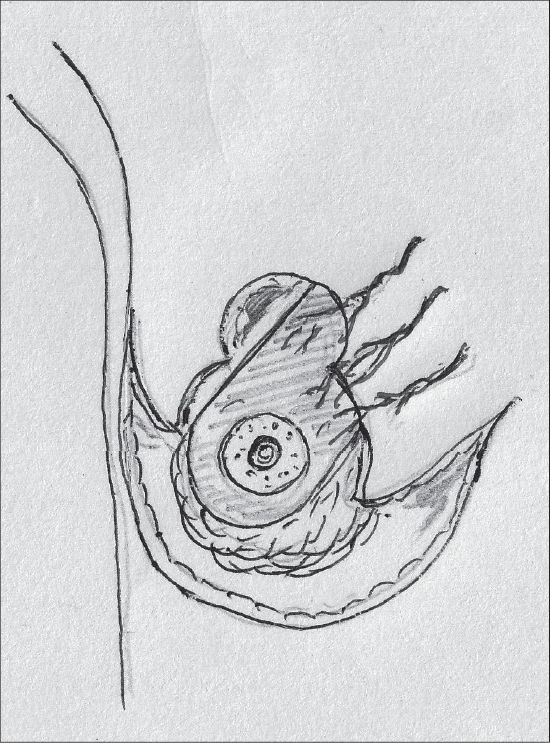
Blood supply of the medial pedicle

Similar to the lateral pedicle, it has become popular following the realization that it has good sensation and good blood supply and can be inset relatively easily [Diagram 5].[37]

## SKIN RESECTION PATTERNS[5][DIAGRAM 6]

**Diagram 6:**
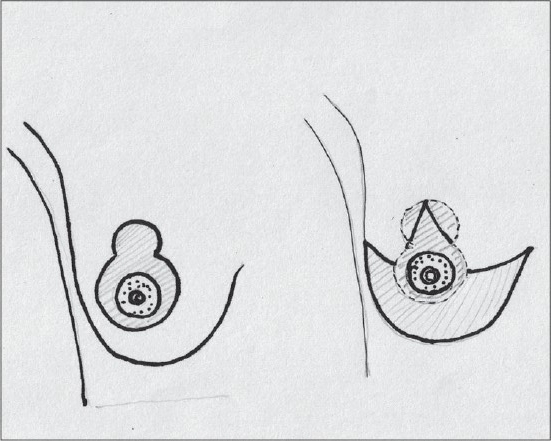
Skin resection patterns

### Inverted T resection (wise pattern)[15,16]

This is best suited for very large breasts and patients who have massive weight loss with skin excess.

It is usually associated with the inferior pedicle but can be combined with other pedicles too.

It is very important to realize that it depends on the skin brassiere to shape the breast.

### Vertical resection

The vertical resection pattern uses the pillars of remaining breast parenchyma to shape the skin as well as hold the breast up. It can be used for a wide variety of cases except the extremely large breasts where it needs some modifications. For larger reduction it may be necessary to add a small T or L to remove excess skin in the inframammary area.

### Circumareolar resection

This method is useful for small resection but still requires a permanent suture.

Use of mesh by Sampaio Goes is a modification but its use is not yet widespread as the mesh is not available everywhere.[38]

### Lateral skin resection

The design avoids the ugly medial scar so often seen in inverted T resections but in larger resections the breasts are displaced medially and are unaesthetic.

The B technique uses a superior pedicle with lateral resection but the design is not very easy to master.[22]

### Pattern with no vertical scar

In this the pedicle is based inferiorly and is broad based.

**Diagram 7:**
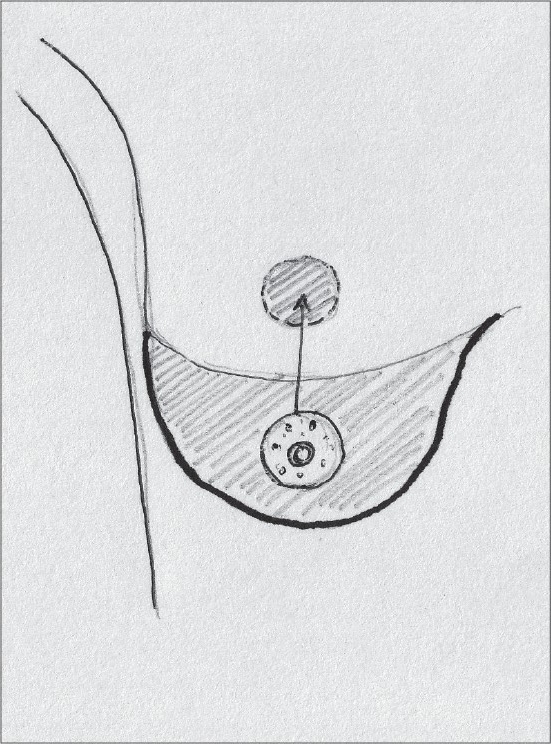
Pattern with no vertical scar

The breast tends to be flat and lacks upper pole fullness. The scar also has a tendency to keep pulling the breast tissue downwards [Diagram 7].[39]

### Liposuction only[26,27]

The procedure is best suited for the fat ptotic breast; it relies on skin elasticity and retraction. It is more likely to preserve nipple sensation and breast feeding potential.

However it is not suited for treatment of gigantomastia in teenagers.

## OPERATIVE TECHNIQUES

### Description of most common techniques (A) Inverted T scar technique Marking the skin resection pattern[40] [Diagram 8]

**Diagram 8:**
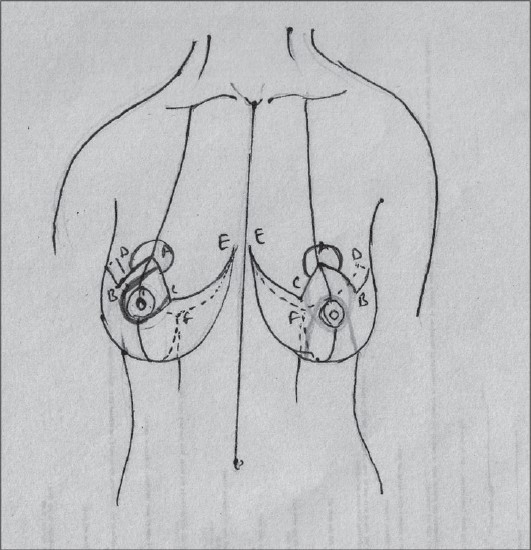
Inverted T resection with all pedicles

The marking for the skin resection is common to all the combinations with superior, medial and inferior pedicle.

**Diagram 9:**
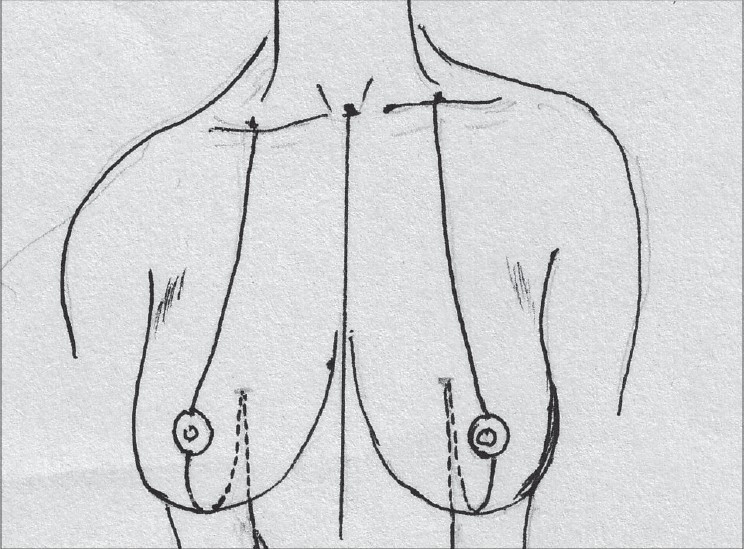
Marking the bony points

With the patient in a sitting position, suprasternal notch, midclavicular points are marked [Diagram 9].

A vertical line joining the suprasternal point to the xiphoid is marked.

The breast meridian is marked next, it may or may not go through the nipple.

**Diagram 10:**
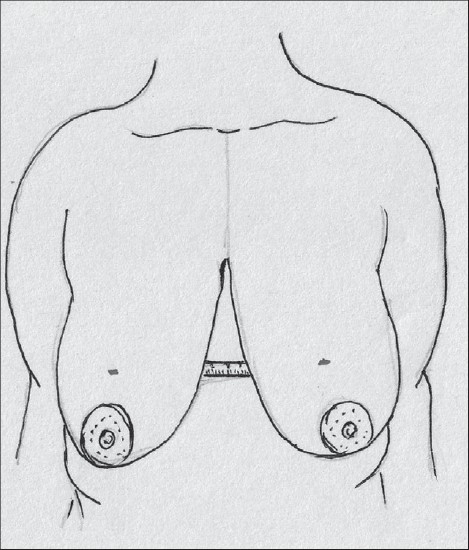
Marking the proposed level of the nipple

**Diagram 11:**
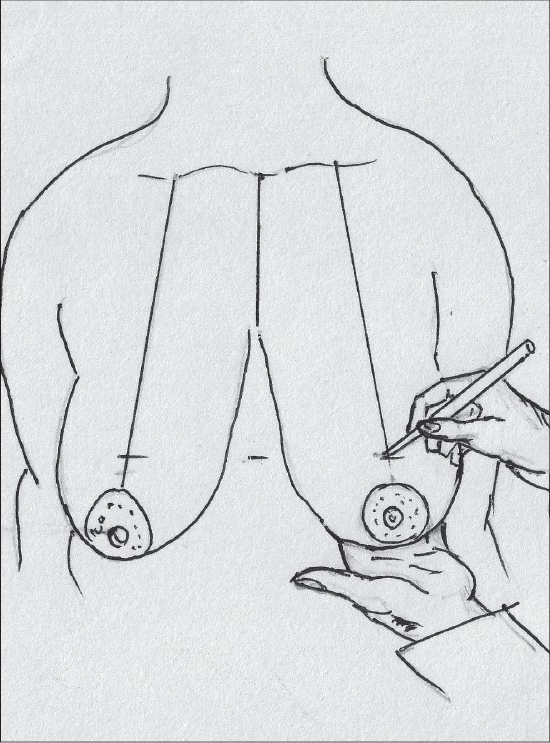
Marking the nipple position

The proposed nipple level is marked by either the Hall Findlay method [Diagram 10] or by the marking of the inframammary crease onto the breast surface by the finger method, this is point A [Diagram 11].

**Diagram 12:**
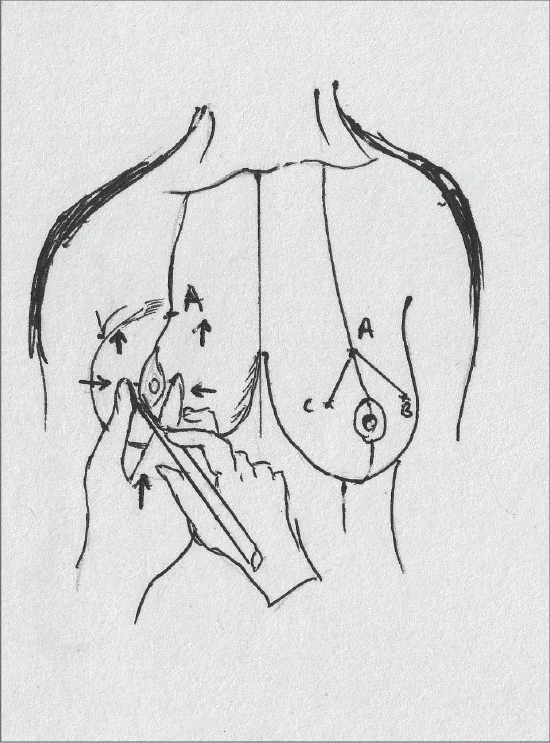
Marking the angles

The angle of the vertical limbs is marked next, by placing the thumb and index finger of one hand and pinching the breast about 6.5 cms from point A [Diagram 12].

The other hand is used to mark the angle points.

**Diagram 13:**
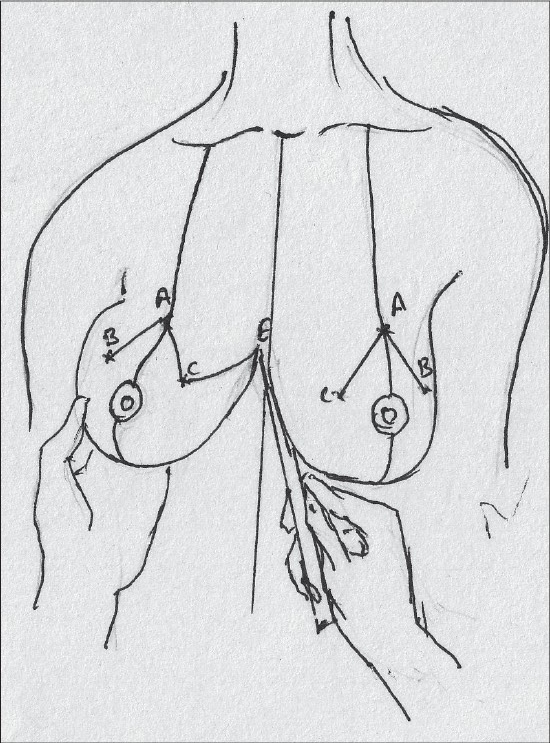
Marking points B and C

These are B (lateral) and C (medial) and they should be equidistant from the breast meridian [Diagram 13].

The angle decides the amount of resection and tightness of closure.

**Diagram 14:**
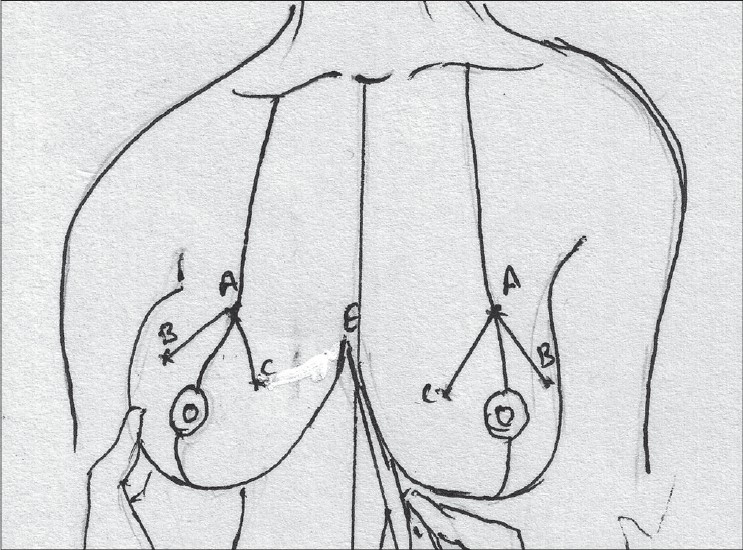
Marking point E

The medial limit of resection (point E) is marked by gently displacing the gland laterally [Diagram 14].

**Diagram 15:**
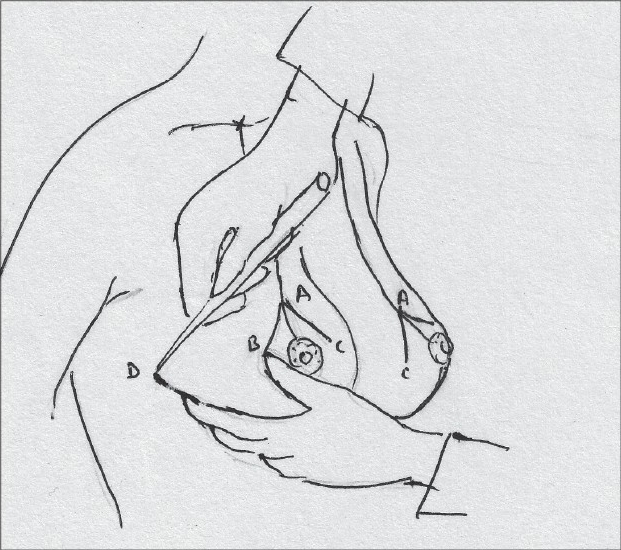
Marking point D

The lateral limit of resection (point D) is marked by gently displacing the gland medially, it should be placed on the gland itself [Diagram 15].

**Diagram 16:**
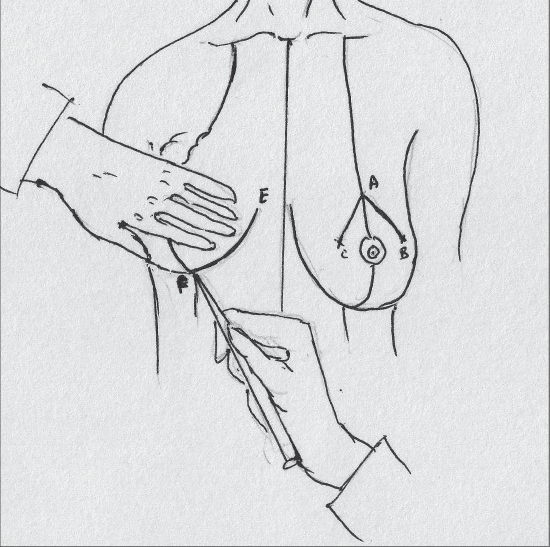
Marking the inframammary crease

The breast is elevated and the inframammary crease is marked, the point where the inframammary crease joins the breast meridian is also marked (point F) [Diagram 16].

**Diagram 17:**
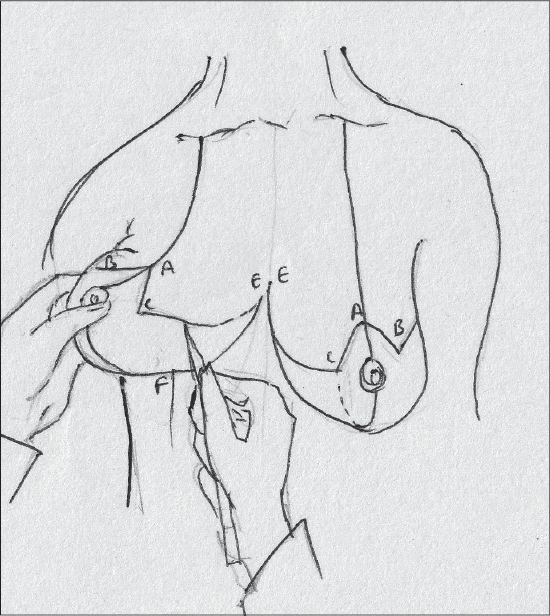
Marking limits of resection

Straight lines are drawn to join points C to E and also from B to D [Diagram 17].

They should be adjusted so that they equal the line from D to E *via* F.

### Inverted T technique with inferior pedicle [Figure 1–2]

**Figure 1:**
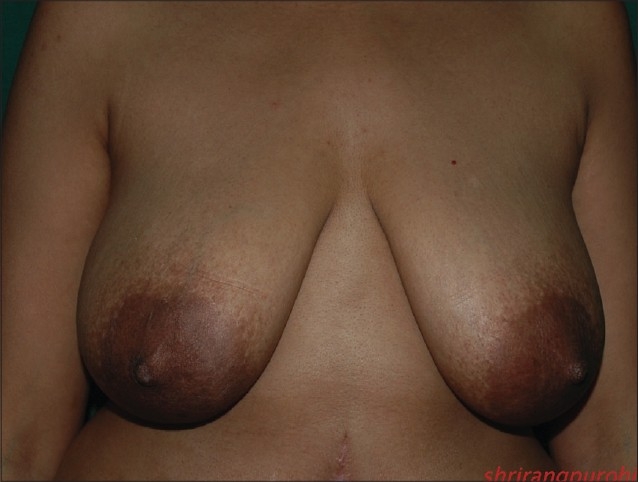
Inverted T resection with inferior pedicle preoperative

**Figure 2:**
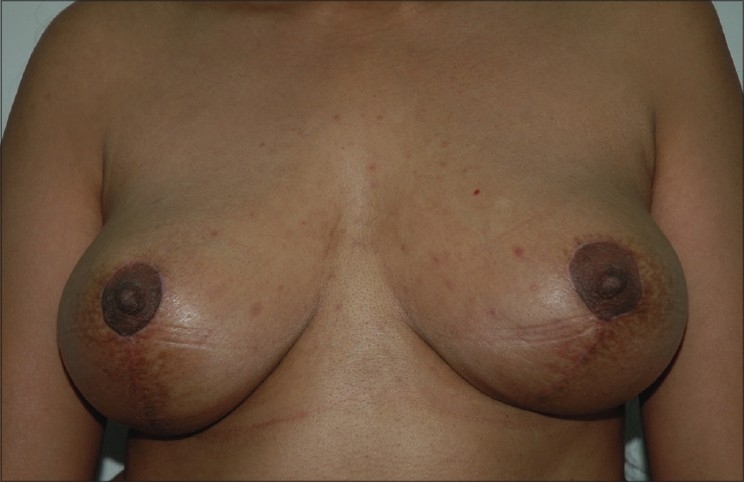
Inverted T resection with inferior pedicle postoperative

The skin resection pattern is marked as described.

**Diagram 18:**
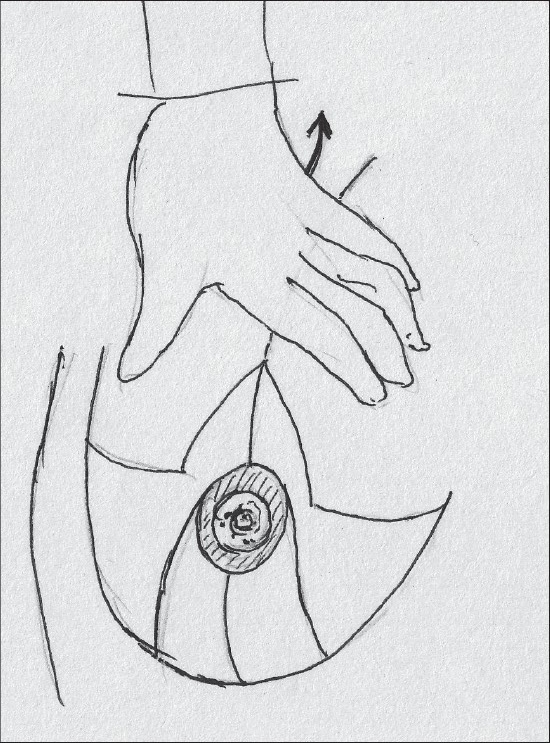
Marking the pedicle

The pedicle is then marked so that the base is 6 to 8 cms wide and centered on the breast meridian, it extends for about 2 cms above the nipple areola complex [Diagram 18].

The procedure begins by stretching the areola and using a cookie cutter to mark the areola so that it is about 3.5 to 4.5 cms diameter. It is then incised to the dermis.

**Diagram 19:**
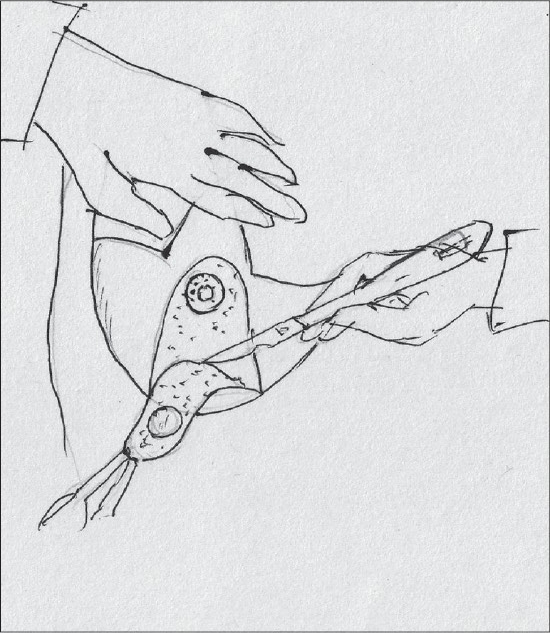
Deepithelialisation of the pedicle

The inferior pedicle is de-epithelialised [Diagram 19].

**Diagram 20:**
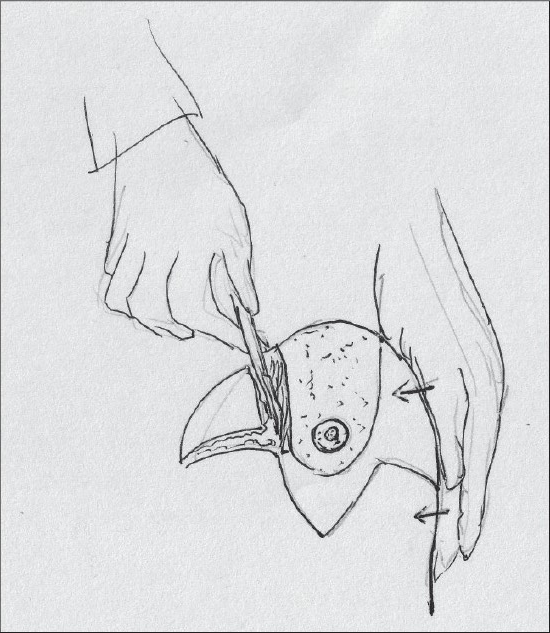
Excision of the breast tissue

With the breast centralized and supported on the chest, medial and lateral triangular excisions are carried out, the lateral being more liberal than the medial [Diagram 20].

**Diagram 21:**
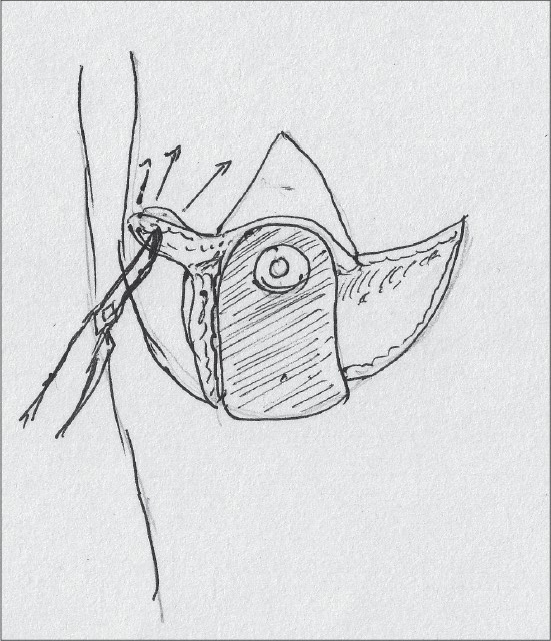
Thinning the superior flaps

The superior flaps are thinned to achieve coning of the breast [Diagram 21].

The superior central excision is carried out between the pedicle and the keyhole margin.

**Diagram 22:**
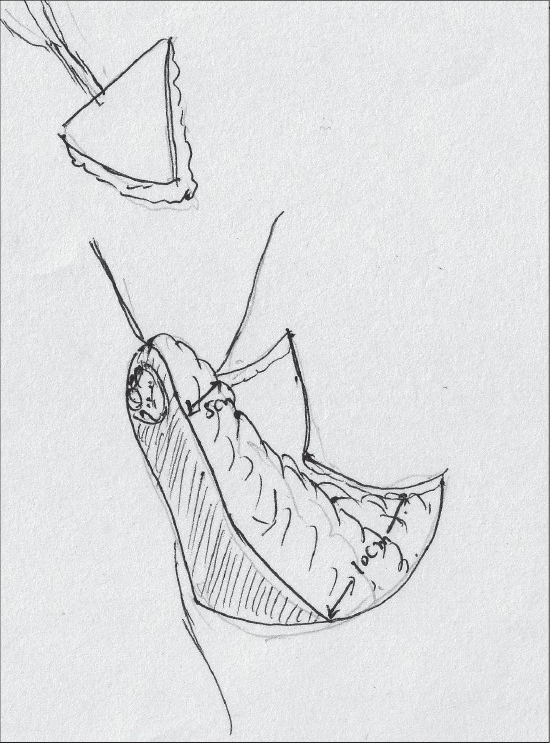
The pedicle

The pedicle should be pyramidal; base should be 10cm wide and tip 5 cm thick [Diagram 22].

**Diagram 23:**
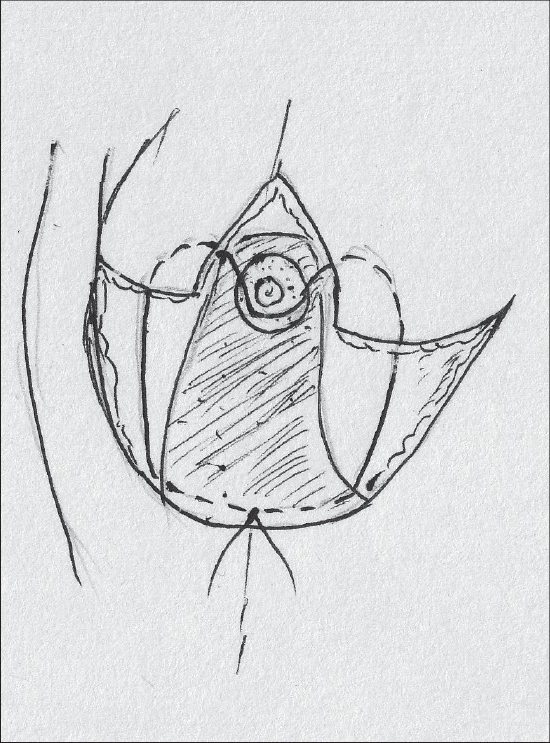
Suturing

After thorough haemostasis, drains are inserted and the medial and lateral flaps are approximated and closed along the inframammary crease [Diagram 23].

**Diagram 24:**
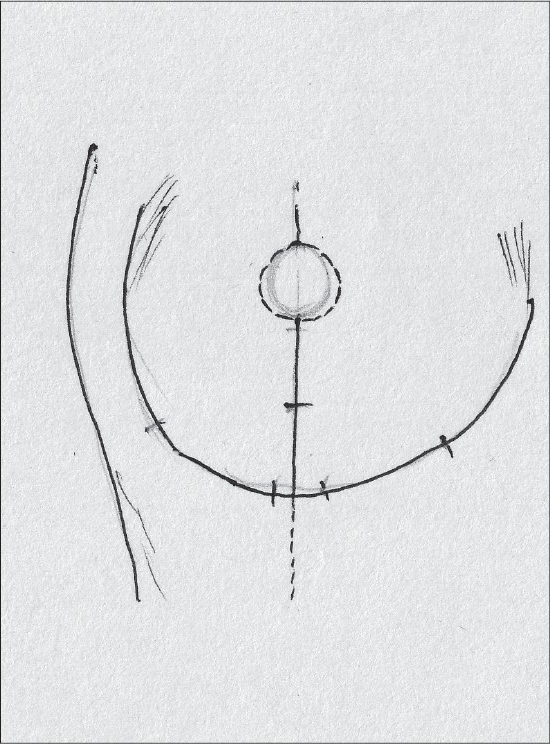
Inset of Nipple Areola complex

An adequate opening for the nipple areola complex is created by excision of skin and the suturing is done with 4-0 monocryl® [Diagram 24].

Closure is performed by using 3-0 monocryl®.

Supporting dressing is given.

### Inverted T technique with medial pedicle [Figures 3–4]

**Figure 3:**
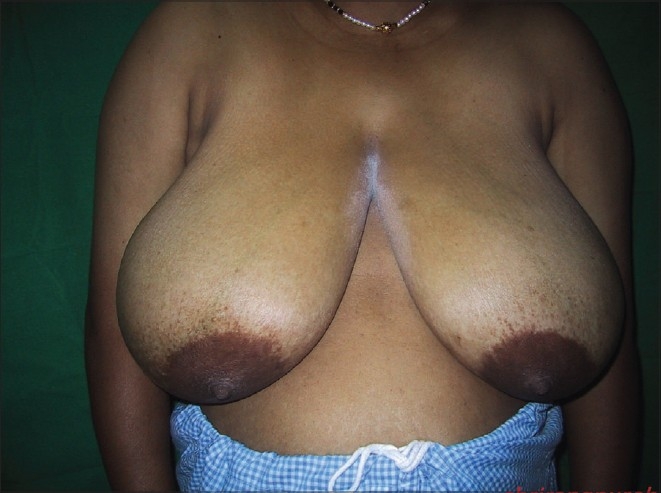
Inverted T resection with medial pedicle preoperative

**Figure 4:**
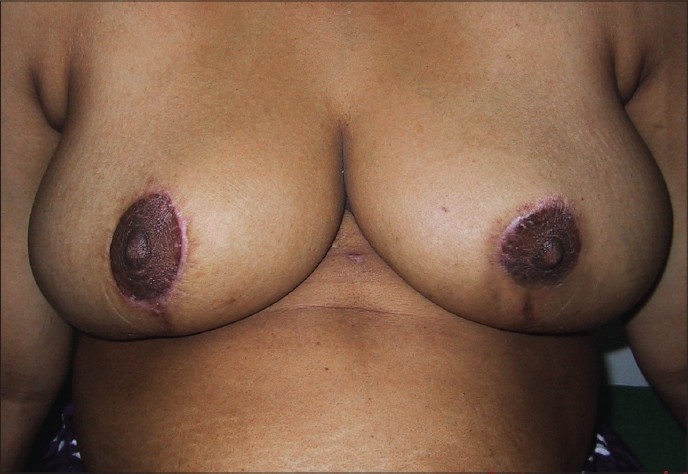
Inverted T resection with inferior pedicle postoperative

Basic markings of the nipple position, inframammary fold remain the same.

The pedicle is then designed by placing it half below and half above the areolar opening and length to width ratio should be 1:1.

**Diagram 25:**
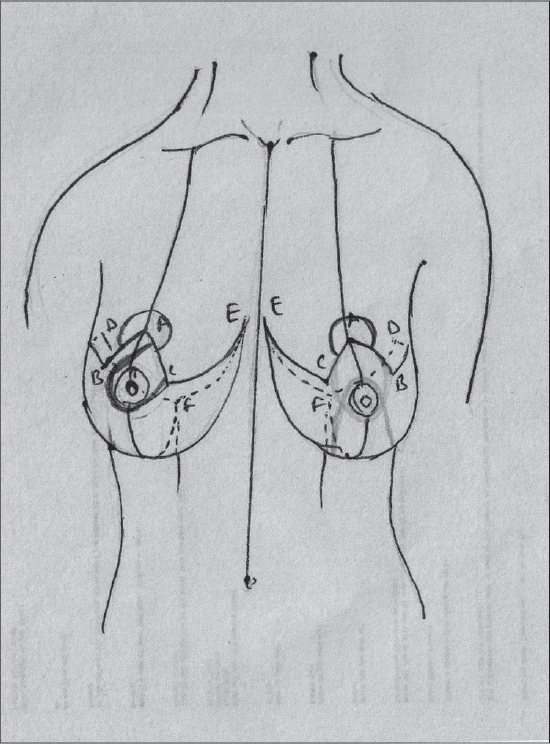
T resection with 3 pedicles

The base is 6.8 or 10 cms, depending on the pedicle length, which has a ratio of 1:1 to the base [Diagram 25-].

After de-epithelialisation of the pedicle, the first incision is made upto the pectoral fascia all around the pedicle and the pedicle is isolated.

Inframammary incision is taken and the area between the pedicle and the crease is resected.

Superiorly the area of areolar inset is resected too. Haemostasis is achieved. Liposuction, if indicated, is performed in the lateral breast area.

First stitch is taken to demarcate the areola opening.

The pedicle is rotated upwards and few stitches are taken to hold it in place.

The breast pillars are approximated to conify the breast.

Drains are inserted and closure is done with 3-0/4-0 monocryl®.

### (B) Vertical short scar techniques Basic manoeuvres

1. Determine the position of the inframammary crease: The method described by Gradinger[41,42] is to place a tape measure under one breast and above the other breast.
2. Hall Findlay[33,34] places the tape under both the breasts and marks the level of the crease in the centre [Diagram 26].

**Diagram 26:**
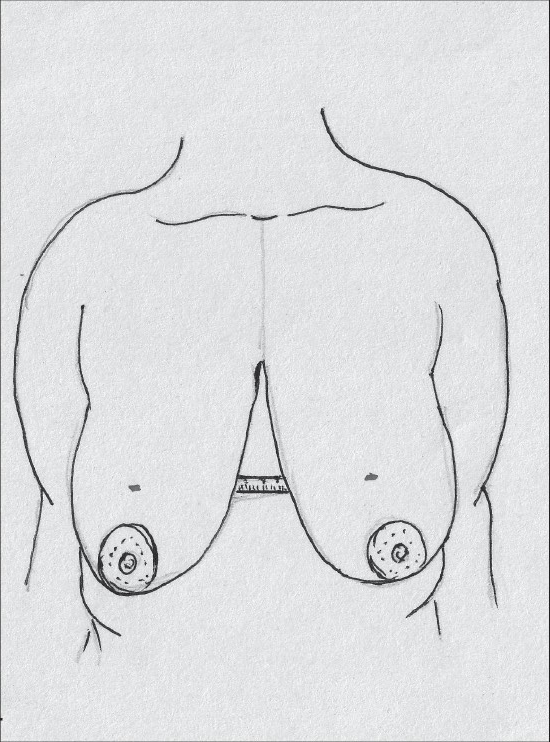
Marking the nipple
3. Mark the breast meridian: Dropping a line from the midclavicular point to the midbreast marks the vertical breast meridian; it may or may not pass through the nipple [Diagram 27].

**Diagram 27:**
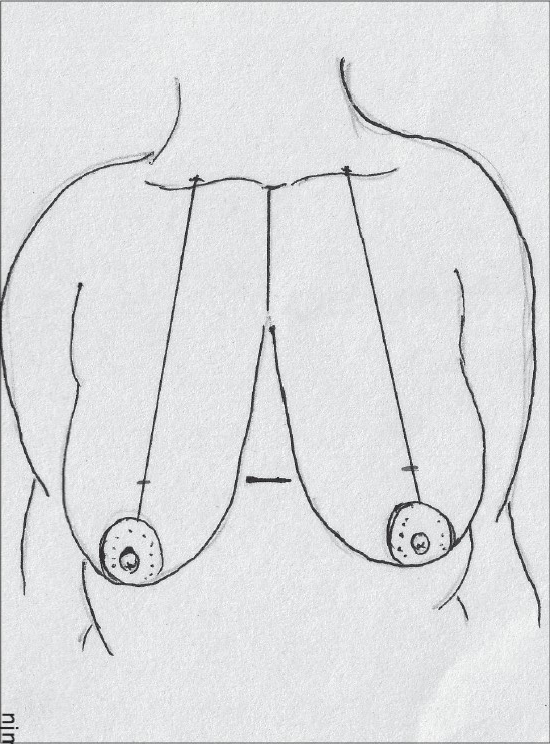
Marking the breast meridian
4. Determine nipple position: The nipple position is marked on the breast meridian at the level of the inframammary crease [Diagram 28].

**Diagram 28:**
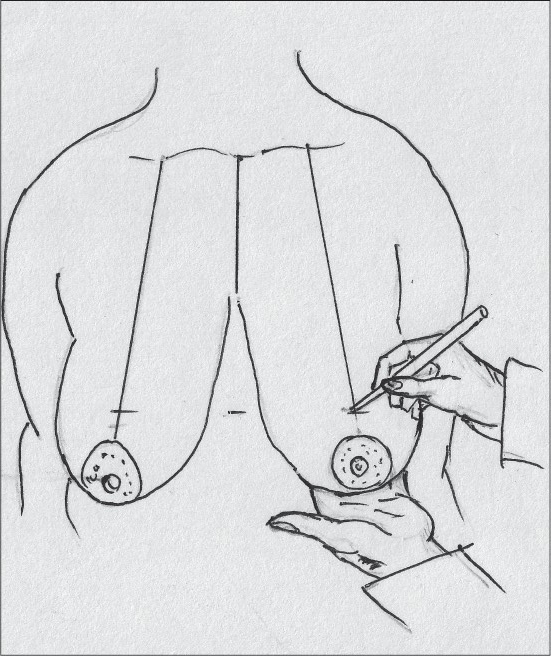
Marking the nipple on the breast meridian
5. Design of the areola: The areola is designed usually to between 2.5 - 4.5 cms in diameter. So the dome-shaped skin opening to receive the areola should be about 14-16 cms. This keyhole can be designed before or after the resection depending on the surgeon's preference [Diagram 29].

**Diagram 29:**
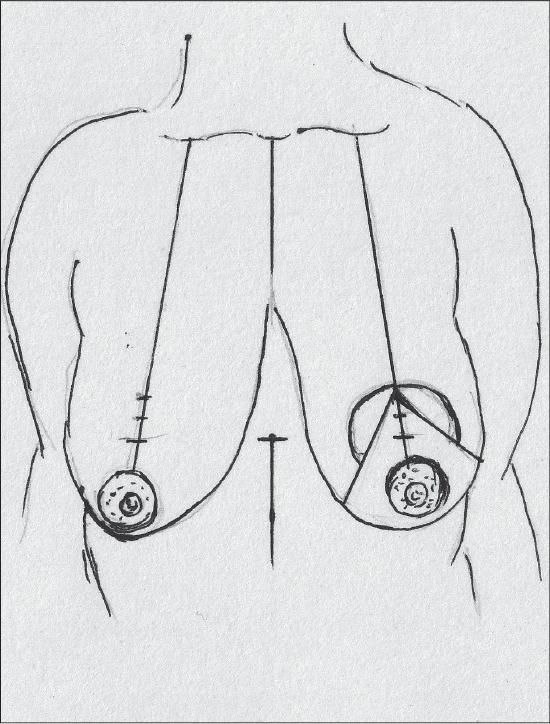
Design of the areola
6. Design of the pedicle: The pedicle can be any of the various choices like superior or medial depending on the patient and surgeon [Diagram 30].

**Diagram 30:**
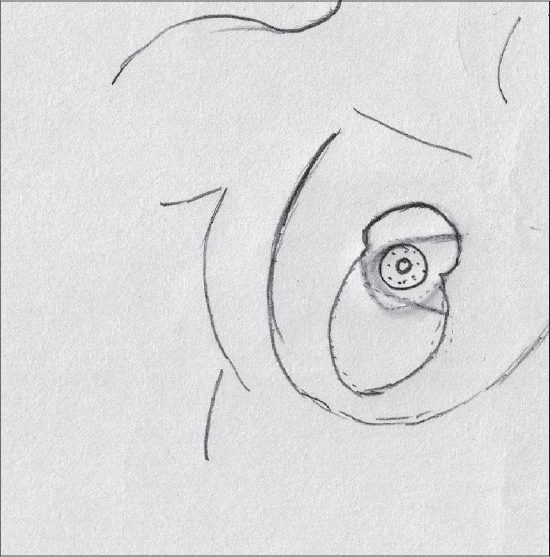
Design of the pedicle

### Vertical short scar technique with medial pedicle [Figure 5–6]

**Figure 5:**
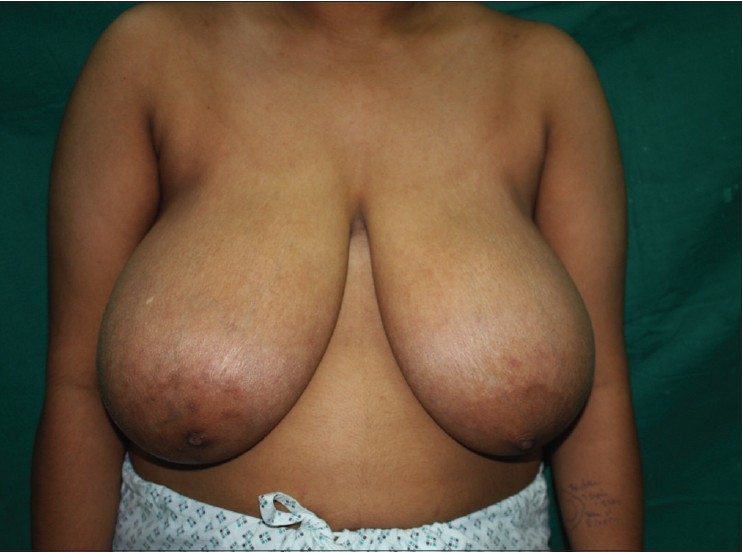
Vertical skin resection with medial pedicle preoperative

**Figure 6:**
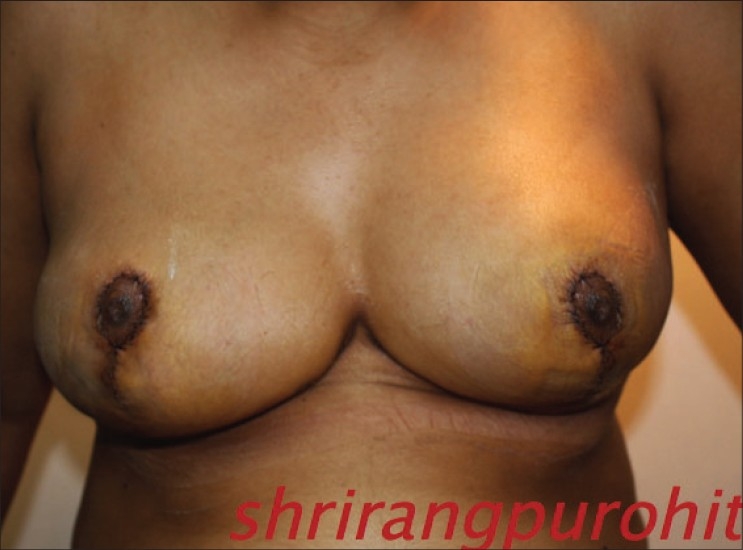
Vertical skin resection with medial pedicle postoperative

The basic markings of breast meridian and inframammary fold are made.

The nipple position on the breast meridian is also marked

**Diagram 31:**
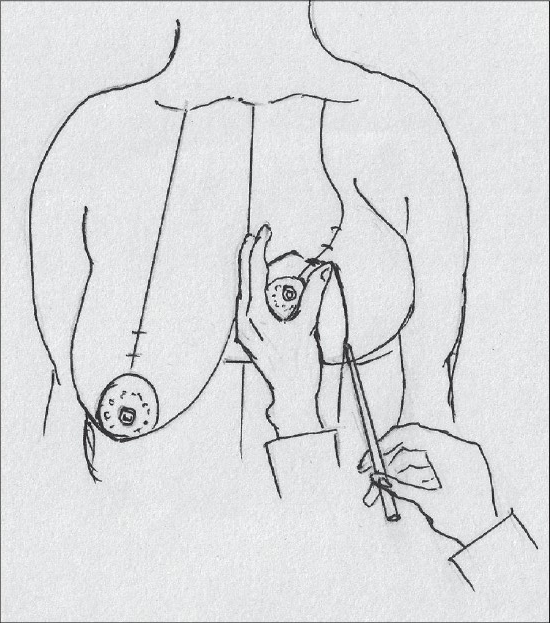
Marking the lateral extent of resection

**Diagram 32:**
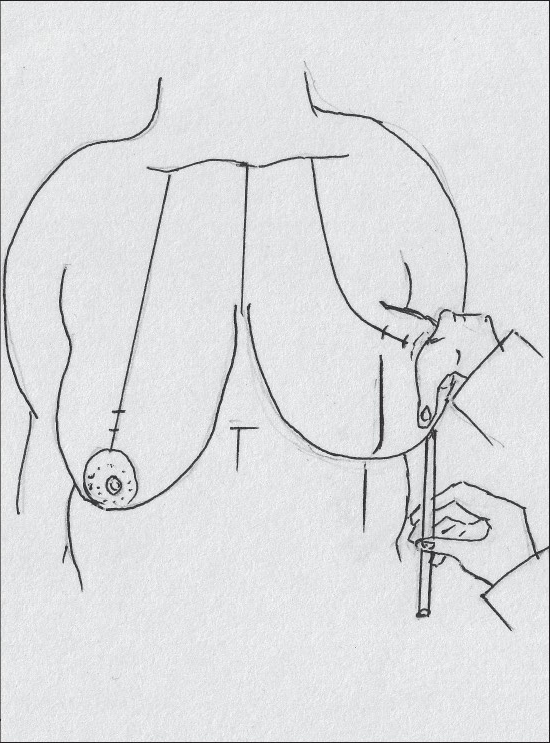
Marking the medial extent of resection

**Diagram 33:**
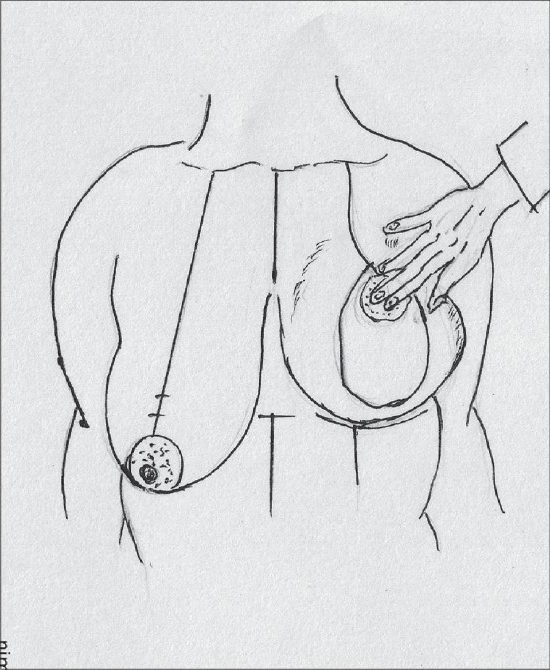
Marking the inferior extent of the resection

The lateral extent of the resection is planned by rotating the breast upward and inward [Diagram 31] and medial extent of the resection is planned by rotating the breast upward and outward [Diagram 32] and lines are dropped on the inframammary crease to join the breast meridian. These two vertical lines are joined to each other well above, at least 2 cms above the inframammary crease [Diagram 33].

**Diagram 34:**
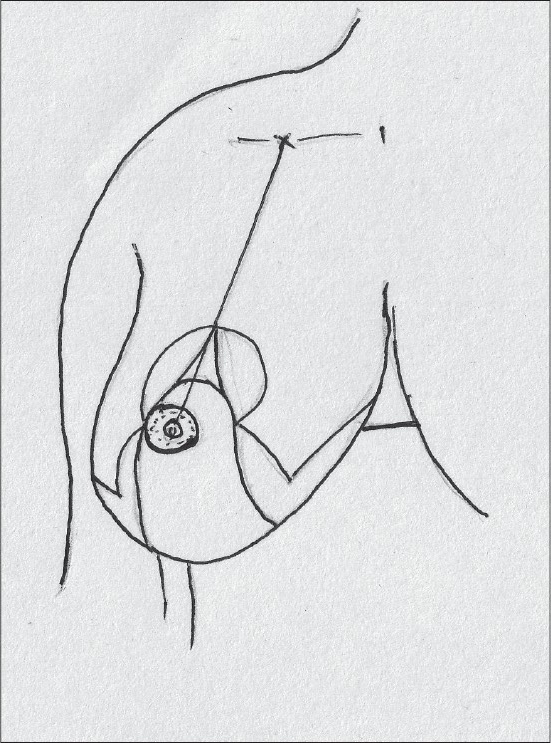
Design of the areola

Superiorly the two lines can be joined as a V, the tip being the point of the nipple, or the areola opening can be designed before starting the surgery [Diagram 34].

The pedicle is then designed by placing it to be half below and half above the areola opening and length and width have a 1:1 ratio.

The base is 6,8 or 10 cms, depending on the pedicle length, which has a ratio of 1:1 to the base.

**Diagram 35:**
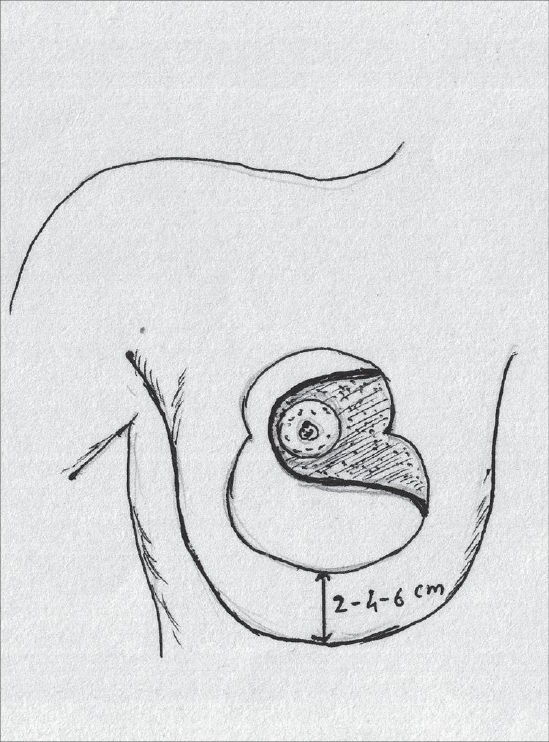
Deepithelialisation of the pedicle

The surgery begins by de-epithelialisation of the pedicle with 1 cm extra margin of skin and tissue [Diagram 35].

The pedicle is full thickness upto the breast wall; it is floppy so as to rotate easily into the opening for the areola.

**Diagram 36:**
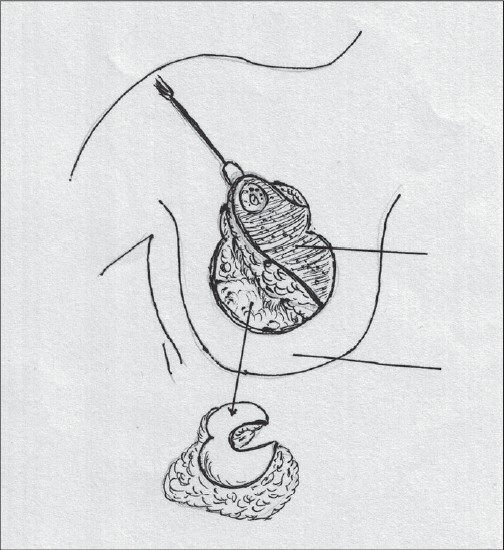
Resection of the breast tissue

Next breast tissue is resected inferiorly, laterally and superiorly [Diagram 36].

**Diagram 37:**
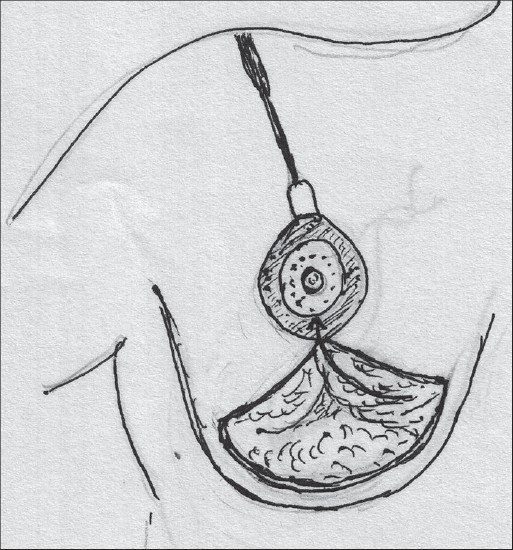
First stitch

After haemostasis, the first suture is at the base of the areola [Diagram 37].

Next the pillars are sutured to give the breast proper shape.

The inferior border of the pedicle becomes the medial pillar.

**Diagram 38:**
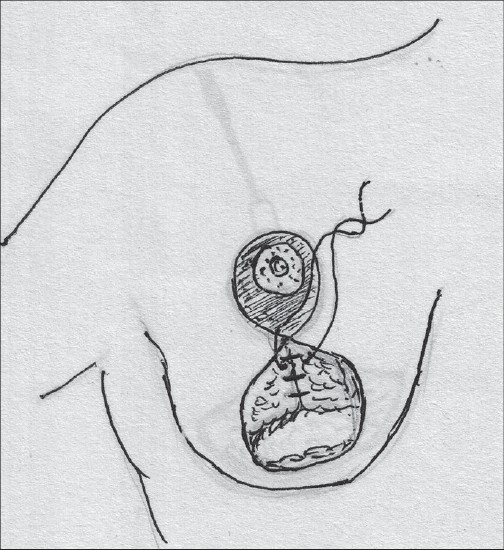
Suturing of the pillars

The pillars are sutured with 3-0 monocryl and should not be more than 7 cm long [Diagram 38].

The areas below the wise pattern are resected medially and laterally below the pillars.

Liposuction is done in areas previously marked usually laterally and in axillary region.

Drains are inserted and closure is done with subcuticular 3-0 /4-0 monocryl®.

### Vertical scar with superior pedicle [Figure 7–8]

**Figure 7:**
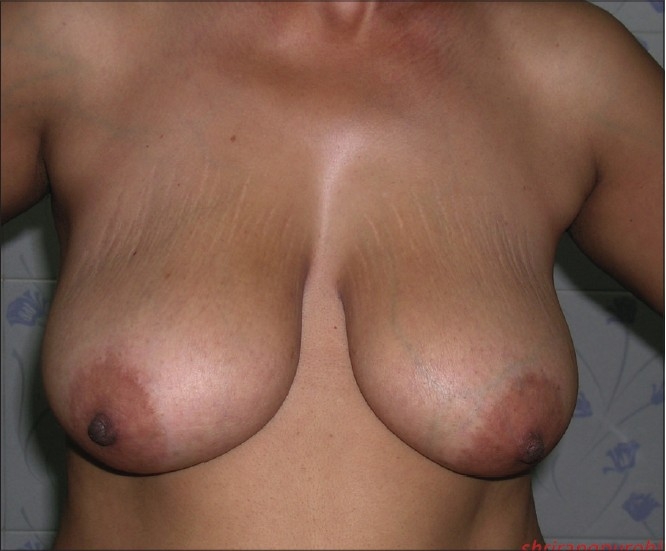
Vertical skin resection with superior pedicle preoperative

**Figure 8:**
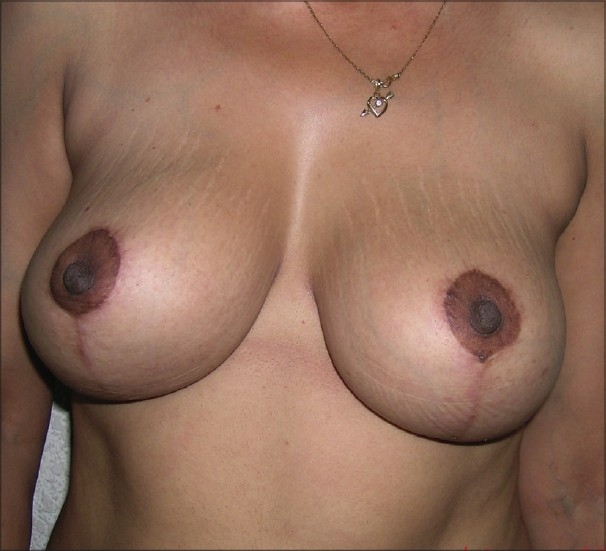
Vertical skin resection with superior pedicle postoperative

All the basic markings remain the same.

The pedicle is marked by first marking the nipple position and then marking the areola opening. The most inferior points of the marking lateral and medial when approximated form a perfect circle. From these two points an arc is marked which includes the areola and has a rim of about 2 cms around it.

The future areola is marked by a cookie cutter after stretching the areola. The area encircled by the markings is then de-epithelialised.

The rest of the procedure is practically the same, except that the resection pattern has a different shape; it is mainly inferior to the areola.

### Liposuction only

This procedure is good for fatty and ptotic breasts, preserves the nipple and areola sensation better and also lactation is better post liposuction. However, it is not suitable for the ideal indication for breast reduction, *viz*. a teenager with large breasts.

### Free nipple areola graft

With the current techniques like Inferior pedicle with an Inverted T skin resection, there is no role for free nipple graft procedure except in selected cases of gigantomastia with ptosis where the pedicle length necessary to translocate the nipple-areola may be enormous.

Even there, the super-flap proposed by Alaa Gheita[43] from Egypt where he has demonstrated safe translocation of a superiorly based Nipple-areolar flap of 40 cms length is available.

## COMPLICATIONS

### Early

1. Haematoma: more common with tumescent type infiltration, careful haemostasis is essential to avoid haematoma.
2. Seroma: Not very common and tend to get absorbed spontaneously over a period of time.
3. Flap Necrosis: More common with inverted T technique, especially in smokers. May require debridement and secondary suturing.
4. Nipple Areola Necrosis: Not very common unless the pedicle is long and patient is a smoker. Very difficult to assess whether intervention is required, it is probably better to let the necrotic area get demarcated and heal than excise and suture.
5. Infection: Not common unless there is vascular compromise, usually due to tight closure.

### Late

1. Asymmetry is a common problem and is usually preoperative asymmetry, which becomes more noticeable later. However attempts should be made to make the breasts symmetrical by proper planning.
2. Lack of proper shape: The vertical skin resection gives a better shape compared to the inverted T resection and also lasts longer.
3. Dog ears: A problem with the inverted T technique, it is difficult to chase dog ears. In the vertical resections the treatment of the dog ear is easier.
4. Under resection: Not very common with the inverted T resections but more likely with vertical resections. May need correction by Liposuction or direct resection.
5. Over resection: Not a common problem. Need counselling rather than surgery. However the occasional case may need augmentation. Ruth Graf procedure where the tissue to be excised is used as a flap to augment the breast primarily is a good option.[44]
6. Unsightly scars: Usually scars settle down over time but in some may bad scar formers the scar can be a problem and have to be dealt with in the usual manner.

### Authors procedure of choice

1. If it is a small breast and the resection is likely to be less than 500 gms then procedure of choice would be a vertical scar reduction with a superior pedicle or medial pedicle.
2. If it is a medium sized breast and the reduction is likely to be more than 500gms then the choice of procedure is vertical scar reduction with medial pedicle.
3. If it is a large breast and the resection is likely to be about 1000gms then the choice would be Inverted T resection and medial pedicle
4. If it is very large breast and the resection is likely to be more than 1500gms then the procedure of choice would be inferior pedicle technique with a inverted T scar.
5. It is of course obvious that the exact amount of resection cannot be predicted and usually one starts getting more and more predictable results from one particular technique and one adapts that to suit the majority of cases, but it is important to master techniques so that the whole range of breast sizes can be covered.

I have now gravitated towards doing medial pedicle technique with a vertical resection or Inverted T resection for majority of the cases that I do and use the inferior pedicle technique with Inverted T resection for the larger breasts.

## CONCLUSION

Breast reduction is a procedure, which has evolved tremendously over the years due to the continuous and ongoing quest on the part of Aesthetic Surgeons to achieve the objective of reducing the breast size, improving breast shape and relocating the nipple areola complex, while minimizing scars and also preserving lactation and innervation to the nipple-areola complex.

Many of the procedures in use currently do achieve all these objectives, but the quest for ideal operation continues.
